# Direct Writing of Nanostructured Metasurfaces by Hot-Electron-Driven Laser Sintering

**DOI:** 10.1021/acs.nanolett.5c04174

**Published:** 2025-10-06

**Authors:** Kai Chang, Kai Wei, Kaushik Kudtarkar, Cagatay Yelkarasi, Ali Erdemir, Shoufeng Lan, M. Cynthia Hipwell, Heng Pan

**Affiliations:** J. Mike Walker ’66 Department of Mechanical Engineering, 14736 Texas A&M University, College Station, Texas 77845, United States

**Keywords:** metasurface, laser sintering, nanocrystal, ligand desorption, hot electron

## Abstract

The precise fabrication of nanoscale metallic structures is pivotal to enabling progress in plasmonics, nanophotonics, and nanoelectronics. In this work, we introduce a high-resolution laser-sintering strategy for facile direct writing of plasmonic metasurfaces, avoiding the need for photolithography or ultrafast laser processing. This method exploits thermally assisted hot-electron-driven desorption and diffusion of aliphatic ligands to facilitate highly localized laser sintering of metal nanocrystals with subdiffraction-limited resolution down to ∼λ/5. A range of functional metasurface nanostructures are demonstrated. A finite-temperature quantum-mechanical model is proposed to predict the superlinear dependence of the ligand desorption rate on laser fluence. This hot-electron-driven sintering method proceeds without inducing the undesired degradation of the ligands, enabling sintering with properties comparable to those of bulk metals. The technique offers promise for the fabrication of polarization-sensitive, wavelength-tunable optical metasurfaces and presents a solution for rapid prototyping of nanodevices.

## Graphical abstract

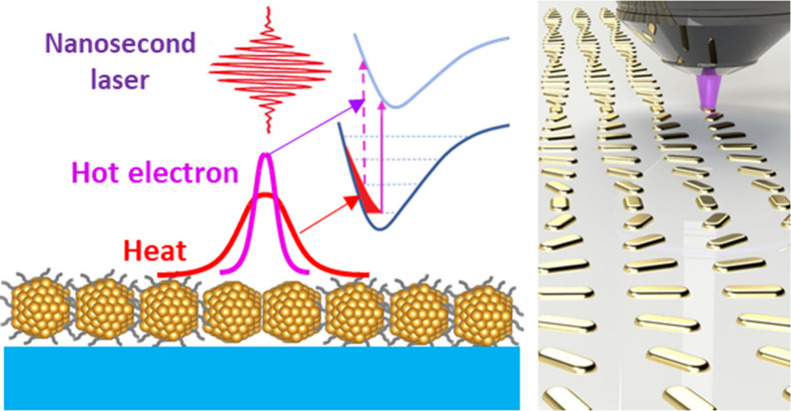

Optical metasurfaces are artificial two-dimensional nanostructures composed of periodic meta-atoms with subwavelength dimensions that manipulate the phase, amplitude, and polarization of light waves.^1^ Metasurfaces attract great attention in the applications of flat lenses,^2,3^ polarization control,^4,5^ holography,^6,7^ wireless communication,^8^ solar energy harvesting,^9,10^ and biomedical imaging.^11,12^

Various techniques have been developed to fabricate high-quality metasurfaces. One major class of these methods is masked lithography, which includes photolithography,^13,14^ nanosphere lithography,^15^ and nanoimprint.^16,17^ Photolithography requires the creation of a mask, followed by pattern transfer and etching. These multistep procedures are time-consuming and costly. While nanoimprinting of nanoparticles successfully reduces process complexity and facilitates direct metal nanopatterning,^18,19^ the fabrication of molds remains a time-intensive step.^20^ Overall, masked lithography methods fall short of meeting the demands for rapid and flexible metasurface fabrication. They are economically intensive due to costly mask fabrication and equipment while also producing chemical waste and a substantial carbon footprint from cleanroom operations. The second class of fabrication methods is maskless lithography, which includes electron-beam lithography,^21,22^ ion-beam lithography,^23^ and optical-based approaches like laser interference lithography^24,25^ and laser direct writing (LDW). Both electron-beam and ion-beam lithographies offer high-resolution patterning capabilities. However, these methods involve complex equipment, and ion beams may cause damage to the fabricated structures.^26^ In contrast, laser interference lithography enables the cost-effective fabrication of periodic metasurface patterns. Nevertheless, its reliance on interference limits its applicability to nonperiodic or arbitrary designs.

LDW, including subtractive processes and additive processes, offers the flexibility and resolution needed for the fabrication of arbitrarily designed nanostructured metasurfaces. Laser ablation is a subtractive approach, creating patterns by selectively removing materials with subwavelength scale resolution.^27–29^ This method is effective in creating negative-relief metasurfaces such as nanohole arrays. Compared with subtractive methods, additive processes offer more versatile options for fabricating positive-relief metasurfaces. Multiphoton polymerization can fabricate metasurfaces with features as small as several hundreds of nanometers.^30–35^ However, it requires ultrafast lasers and has limitations in materials.^36^ Another additive method is laser-induced reduction in which metal precursors are photochemically reduced to metallic form under laser exposure. This technique enables the generation of metallic patterns with feature sizes on the order of several hundreds of nanometers,^37,38^ although the photoreduction reaction may require tens of seconds to form a single unit cell of the metasurface, resulting in a relatively slow processing speed. Laser-induced crystallization involves transforming localized regions of a phase-change material (PCM) from the amorphous to crystalline state using a laser, thereby generating spatially resolved variations in the physical properties between the two phases.^39–41^ This approach is limited to PCMs such as Ge_2_Sb_2_Te_5_ or In_3_SbTe_2_, which restricts the range of applicable materials for metasurface fabrication. Laser sintering is an additive approach and applicable to a broad range of materials including polymers,^42^ ceramics,^43^ and metals.^44,45^ Typically, continuous-wave (CW) laser sintering achieves micron-scale features^46–49^ due to its reliance on the thermal sintering mechanism^50,51^ in laser–nanoparticle interaction. In contrast, achieving nanoscale sintering generally requires ultrafast lasers,^52–56^ which deliver pulses with extremely high peak intensities to induce nonlinear absorption while minimizing thermal effects^57,58^ or requires near-field optics.^59^ Although laser sintering based on ultrafast lasers is promising for metasurface fabrication, their use can be cost-prohibitive for large-scale production.

In this work, a high-resolution laser-sintering strategy based on cost-effective lasers for facile direct writing of plasmonic metasurfaces is reported. It enables direct printing of metals with down to ∼70 nm line width without the use of ultrafast lasers or light-responsive inks. A range of functional nanostructured metasurfaces has been successfully demonstrated. A laser-driven photochemical reaction model, derived from a quantum-mechanical ligand desorption model, is proposed to predict experimental outcomes and validate the underlying mechanism.

The basic concept of subdiffraction-limited laser sintering is illustrated in Figure 1a. In this study, a 355 nm nanosecond laser is focused onto the spin-coated nanocrystal films through the back side (BS) of the transparent substrate. Alternatively, front-side (FS) irradiation can be employed for both transparent and opaque substrates (see section A in the Supporting Information for details). The laser wavelength and pulse duration are chosen to achieve highly localized nonthermal (electronic) excitation with minimal heat diffusion (Figure 1a). The ultralow laser power level (∼a few microwatts) is sufficient to induce nanocrystal fusion and sintering. The process enables the direct writing of functional micro- and nanoscale patterns with subdiffraction-limited features. Figure 1b shows examples of fabricated metasurfaces, including concentric antennas, phase-shifting antennas, chiral structures, and bowtie antennas. These structures enable control over near-field coupling,^60^ induce phase shifts,^61^ and provide chiral-optical resonances^62^ and localized resonance fields.^63^

**Figure 1:**
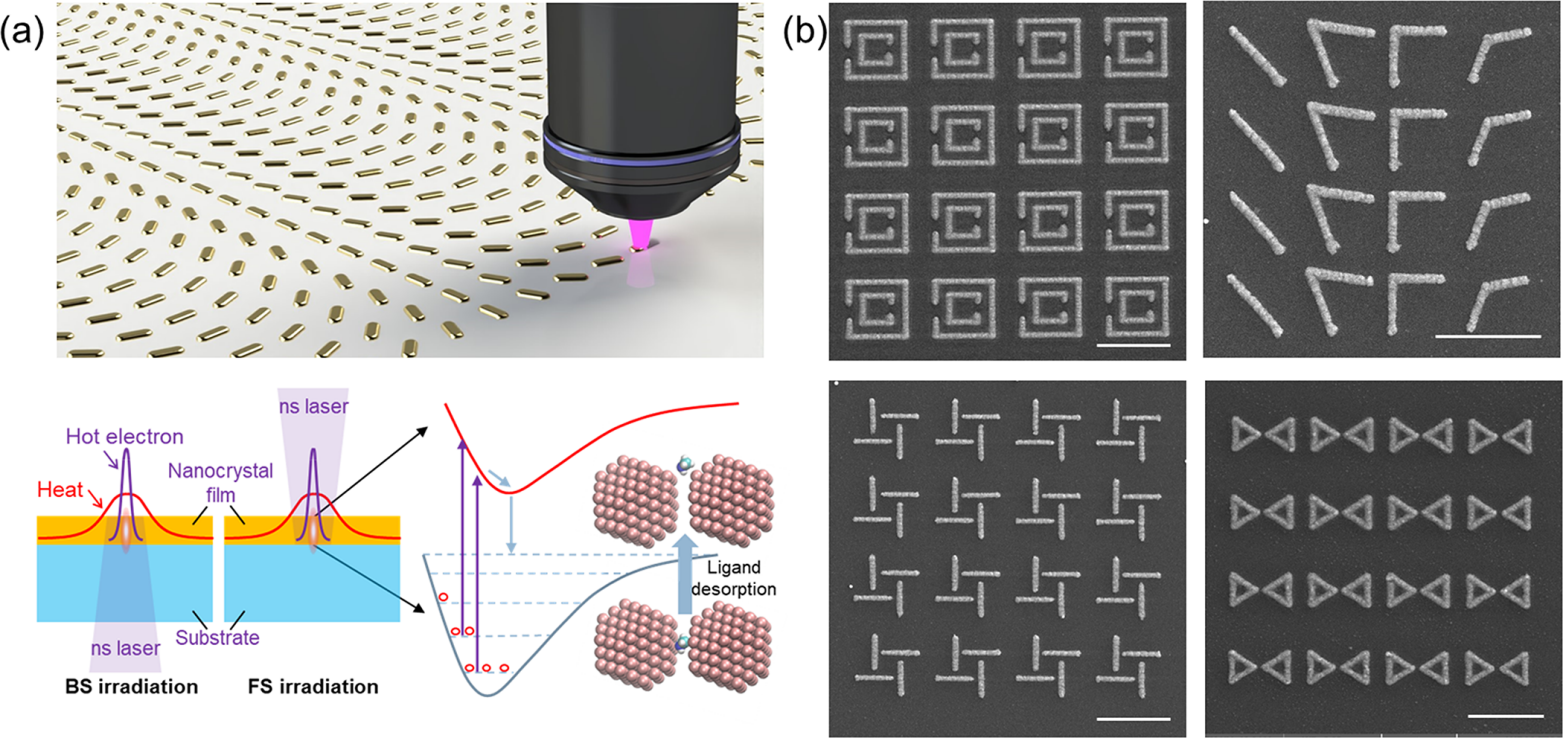
Subdiffraction-limited hot-electron-driven laser sintering. (a) Schematic of subdiffraction-limited hot-electron-driven laser sintering of nanocrystals for metasurface fabrication. (b) Fabricated nanostructured metasurface containing concentric antennas, phase-shifting antennas, chiral plasmonic antennas, and bowtie antennas. The scale bar in part b is 2 μm.

A series of horizontal lines was fabricated by using various laser fluences and scanning speeds. As shown in Figure 2a, the line width decreases with reducing laser fluence. The smallest line width as fabricated is ∼70 nm (∼λ/5), confirming subdiffraction-limited laser-sintering capability. Figure 2b shows the dependence of line width *d* on the laser fluence and laser scanning speed, which can be modeled based on the following laser-driven photochemical reaction model (also shown in eq 9 in the Supporting Information):

1 $$d=\omega_{0}\sqrt{\frac{2}{n}\;\ln\left(\frac{{C_{t}\omega_{0}\left(2.3F\right)}^{n}}{v}\right)}$$

Here, ω_0_ is the radius of the focused laser spot (nm), *F* is the laser fluence (mJ/cm^2^), *v* is the laser scanning speed (μm/s), and *C*_t_ is a parameter indicating the sintering threshold. The relationship between *d* and *F* is fitted using the model, from which the parameters ω_0_, *C*_t_, and *n* are extracted (see the Supporting Information). The fitting results yield 2ω_0_ = 442 nm and *n* = 3.59. The fitted spot size 442 nm is comparable to the diffraction-limited spot size of a circular and uniform beam 1.22λ/NA = 320nm. The exponent *n* > 1 signifies a superlinear dependence of the laser-driven photochemical reaction rate on fluence, following a ∼*F* ^*n*^ relationship. The relationship between the scanning speed *v* and laser fluence *F* is shown in the inset of Figure 2b. To achieve a line width of ∼150 nm, higher laser fluence is required as the scanning speed increases, with speeds up to 500 μm/s successfully demonstrated.

**Figure 2:**
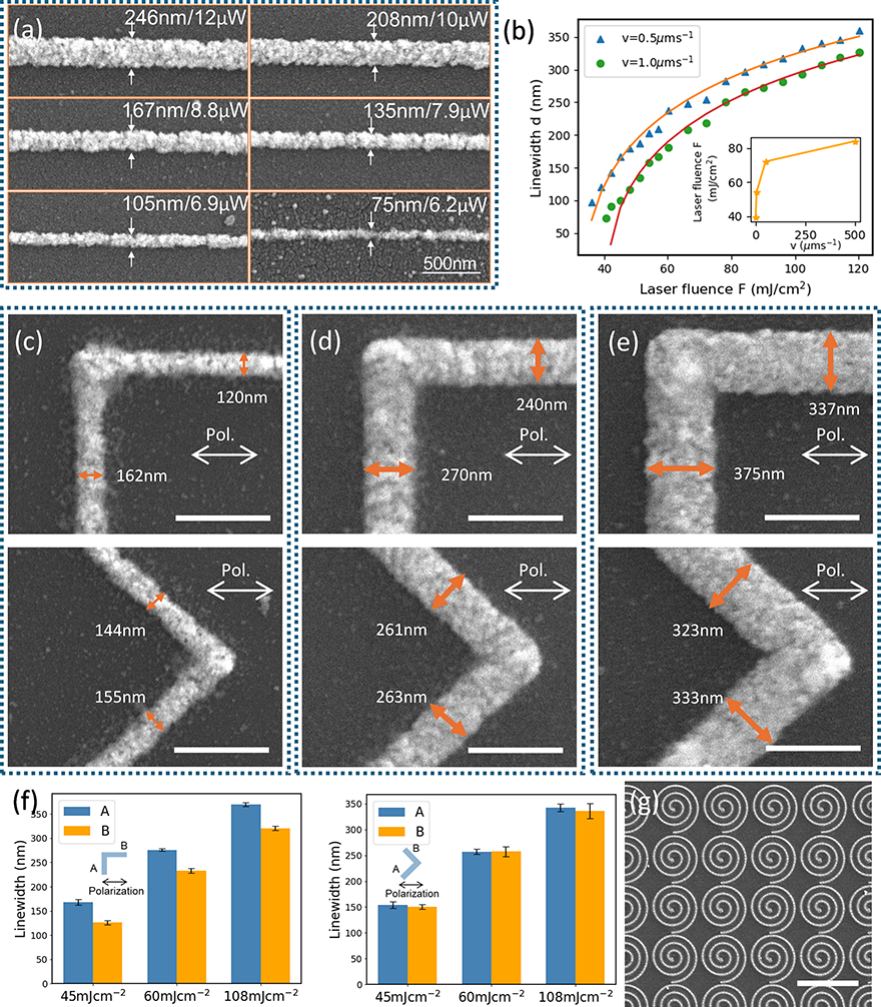
Dependence of the laser-sintered line width on the laser fluence and polarization. (a) Nanoline patterns with the nanoscale line width fabricated by a laser power at microwatt levels. (b) Dependence of the line width on the laser fluence and dependence of the laser scanning speed on the laser fluence (inset). (c–e) Nanoline patterns sintered with the polarization direction perpendicular, parallel, or at 45° or 135° relative to the laser scanning direction at laser fluences of (c) 45, (d) 60, and (e) 108 mJ/cm^2^. (f) Summary of the line width fabricated with different polarizations. (g) Fabricated spiral nanostructures with a circularly polarized beam. The scale bar in parts a and c–e is 500 nm, and the scale bar in part g is 5 μm.

It is found that the polarization of the beam has profound effects on the line width of the laser-sintered lines. Line sintering with the polarization direction perpendicular, parallel, or at 45° or 135° relative to the laser scanning direction was fabricated and is shown in Figure 2c. Line patterns were fabricated under identical power and scanning speed conditions, with varying polarization. Line features written with the linear polarization parallel to the scan direction exhibited narrower line widths compared to those written with perpendicular polarization. When using higher laser power to obtain a larger line width, in Figure 2d,e, the polarization-dependent line-width difference becomes more evident. Such a phenomenon is caused by the electric-field-intensity concentration in the polarization direction resulting in directionally determined intensity distribution and larger line width. Meanwhile, the 45° and 135° lines show almost equal line widths, and the line width is between lines fabricated with perpendicular and parallel polarizations. A similar result was reported in a laser ablation study.^27^ The polarization dependence on the line width is summarized in Figure 2f. When the printing resolution is considered, the polarization effect needs to be considered. To minimize the anisotropic effect induced by polarization, the laser beam was modulated with a 1/4 waveplate to generate circular polarization. Figure 2e shows the scanning electron microscopy (SEM) image of the spiral structures fabricated with the circularly polarized beam exhibiting significantly more uniform lines in both the horizontal and vertical directions.

Raman spectroscopy measurement was performed to elucidate the evolution of the ligands during the laser-sintering process. Figure 3a illustrates the Raman spectra of nanocrystal films annealed by conventional thermal sintering; the inset shows the visual appearance from optical reflectance. The Raman spectra of the as-deposited film show characteristic peaks around 2800–3000 cm^–1^, corresponding to C–H vibrations in oleylamine.^64^ Thermal sintering at 200 °C imparts a reflective appearance to the film, while strong peaks at approximately 1340 and 1590 cm^–1^, corresponding to the D and G bands of graphitic carbon, respectively, appear in the Raman spectra (Figure 3a). These bands are attributed to carbonaceous byproducts formed during ligand decomposition.^45,65^ The carbonaceous byproducts can be further removed by subjecting them to thermal sintering at 350 °C in ambient air^65^ (Figure S3b). In contrast, laser sintering with 35 and 70 mJ/cm^2^ laser fluences produces a highly reflective film comparable to that obtained by thermal sintering, while the D and G bands are largely absent in the relevant range, suggesting a different sintering mechanism (Figure 3b). It is postulated that incident photons (3.5 eV) generate energetic hot electrons in gold nanocrystals, which subsequently scatter with the ligand, leading to ligand desorption and diffusion that facilitate the nanocrystal sintering. In the hot-electron-driven process, nanocrystal fusion and sintering are accomplished by ligand desorption and diffusion (Figure 3c), while thermal decomposition of ligands associated with the formation of D and G bands that occur in conventional sintering (Figure 3a)^65^ is largely avoided. To further validate the photoexcited hot-electron-driven nature of the sintering process, a laser operating at 1064 nm with identical pulse duration and repetition rate was used for comparison. No sintering was observed, and strong D and G bands emerged (Figure S4), indicating significant carbonaceous residue.

**Figure 3:**
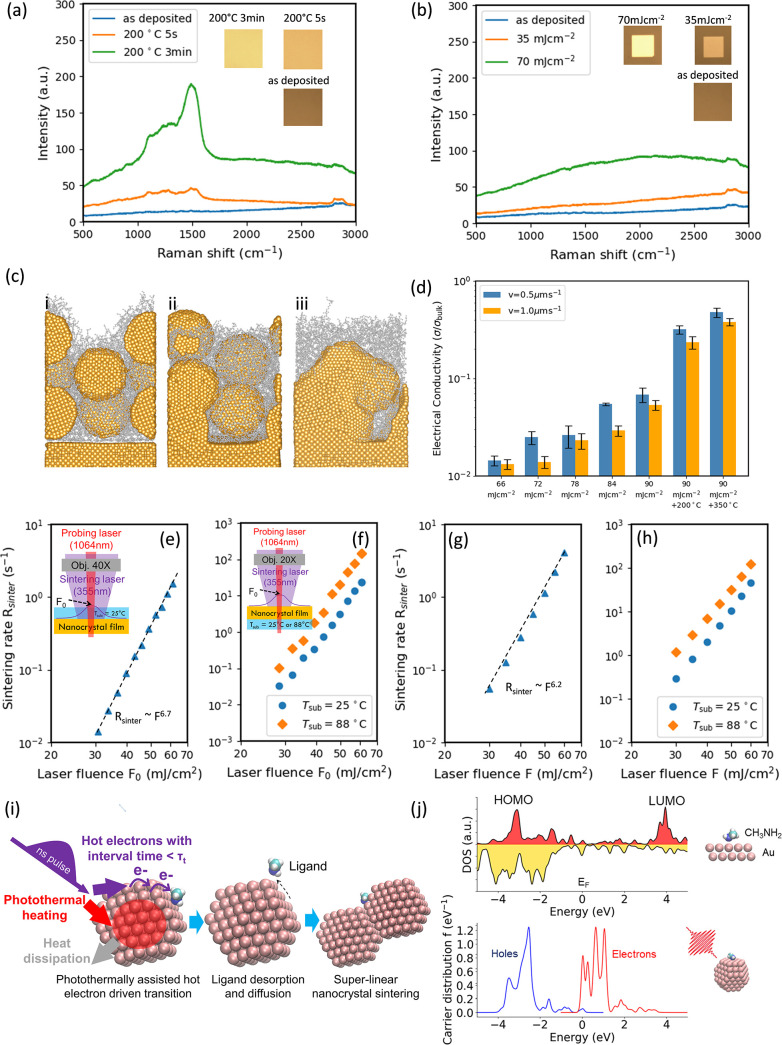
Characterization of laser-sintered films and the sintering mechanism. Raman spectra of (a) as-deposited nanocrystal films and (b) laser (355 nm)-sintered nanocrystal films. (c) Illustration of the nanocrystal fusion and sintering process mediated by ligand diffusion and desorption. (d) Electrical conductivity of laser-sintered nanocrystal films. (e) Superlinear dependence of the laser-sintering rate on the laser fluence. (f) Thermally assisted hot-electron-driven sintering, as is evident by the increase in the sintering rate with increasing substrate temperature. (g) Quantum-mechanical model-predicted superlinear dependence of the laser sintering rate on the laser fluence. (h) Quantum-mechanical model-predicted dependence of the sintering rate on the temperature. (i) Illustration of the transport phenomena during the thermally assisted hot-electron-driven laser sintering process. (j) Total density of states of the methylamine–gold complex and partial density of states of the methylamine. Calculated energy distribution of hot carriers in the nanocrystal.

Ligand desorption and diffusion promote the formation of long-range conductive internanocrystal networks, leading to ∼1–10% of the bulk electrical conductivity as measured by the nanoelectrode fabricated by laser sintering with a fluence of 66–90 mJ/cm^2^ (Figure 3d). It is worth noting that this level of electrical conductivity was achieved despite the presence of residual ligands in the films that are not degraded by laser irradiation. These residual ligands can be removed through subsequent thermal decomposition, if necessary. This is evidenced by the further improved electrical conductivity of laser-sintered films subjected to additional thermal sintering, which reaches up to ∼50% of the bulk conductivity, comparable to that of films sintered exclusively by thermal means (Figure 3d). Further support comes from Raman spectroscopy, where the spectra of laser-sintered films treated with additional thermal sintering at 200 and 350 °C show ligand evolution similar to that observed in films sintered purely thermally (Figure S3). These results indicate that hot-electron-driven laser sintering, in conjunction with optional thermal sintering, enables excellent patterning resolution while maintaining sintering quality.

To characterize the kinetics of the hot-electron-driven ligand reaction process, an *in situ* transmission measure was conducted (Figure S1). First, transmission measurement was performed without externally heating the substrate (*T*_sub_ = 25 °C). The relationship between the laser fluence and sintering rate is shown in Figure 3e. The sintering rate exhibits a power-law dependence on the laser fluence with an exponent of *n* ∼ 6.7. Second, the sintering rate measurements were conducted at elevated substrate temperature (*T*_sub_ = 88 °C) and compared with the sintering rate at *T*_sub_ = 25 °C. Figure 3f demonstrates that the substrate temperature enhances the sintering rate by approximately 3–6 times, thereby supporting a thermally assisted, hot-electron-driven sintering mechanism.

The observed superlinear dependence in Figure 3e suggests that the interval time between successive hot-electron-driven events becomes shorter than certain characteristic relaxation time τ in photoexcited nanocrystal films.^66^ At high laser intensity, desorption induced by multielectron transitions (DIMET) is possible wherein the interval time between hot electrons is shorter than the vibrational relaxation time τ_vib_ (∼a few picoseconds).^67,68^ However, the laser intensity applied in this study is unlikely to drive the interval time below τ_vib_. Another relevant characteristic time in photoexcited nanocrystal films is the heat dissipation time τ_t_ (∼500 ps to a few nanoseconds) due to heat dissipation to the substrate.^66^ Heat generated in the nanocrystal from the preceding hot-electron excitation elevates the nanocrystal temperature. Before the thermal energy dissipates over time τ_t_ , the increased probability of the ligand occupying higher vibrational energy states lowers the activation barrier for subsequent hot-electron-induced reactions.^69^

Based on this analysis, the microscopic mechanism of the thermally assisted hot-electron-driven sintering is illustrated in Figure 3i. During the laser pulse of ∼20 ns, a flux of photoexcited hot electrons is generated, which scatters with the ligands, transferring quanta of vibrational energy to induce ligand desorption. These hot electrons are thermalized, followed by the electron–phonon relaxation, which raises the temperature of the nanocrystal, which, in turn, assists the subsequent hot-electron-driven desorption processes. To compute the ligand desorption rate driven by hot electrons, a quantum-mechanical desorption model is developed (see the Supporting Information), wherein the oleylamine molecule is modeled as a methylamine molecule absorbed on the gold (111) surface. Hybridization of the LUMO level of the methylamine with gold atoms forms states ∼4.1 eV above the Fermi level (Figure 3j). According to time-dependent density functional theory (TDDFT) calculations, hot electrons with energies up to ∼3.5 eV above the Fermi level are transiently formed in the nanocrystal, as shown in Figure 3j. Transient hot electrons engage in nonadiabatic coupling with the hybridized amine–gold states, inducing vibrational excitation and driving amine desorption. The reaction rate coefficient *k* of the ligand desorption on the nanocrystal can be written as

2 $$k=\frac{J}{\#}\int_{E_{F}}^{\infty }R\left(\varepsilon ,T\right)\;f\left(\varepsilon \right)\;d\varepsilon$$

wherein *J* is the flux of hot electrons in a nanocrystal, # is the number of ligands per nanocrystal, $\int_{E_{F}}^{\infty }R\left(\varepsilon ,T\right)\;f\left(\varepsilon \right)\;d\varepsilon$ is the probability of ligand desorption per photoexcited hot electron,^68^ and *f*(ε) is the energy distribution of photoexcited hot electrons (Figure 3j). Figure S6 shows the calculated desorption probability $\int_{E_{F}}^{\infty }R\left(\varepsilon ,T\right)\;f\left(\varepsilon \right)\;d\varepsilon$ induced by a photoexcited hot electron. Temperature significantly enhances the desorption probability, increasing it by 3–4 orders of magnitude as the temperature rises from 300 to 800 K. The hot electron flux per nanocrystal *J* can be computed by TDDFT and is shown in the inset of Figure S6. At a laser fluence of ∼10–100 mJ/cm^2^, the hot electron flux per nanocrystal is found to be 8 × 10^8^–8 × 10^9^/s corresponding to a 1.25 ns to 125 ps interval time between hot electrons. The interval time is comparable to or shorter than the heat dissipation time, τ_t_ , supporting a thermally assisted mechanism driven by hot electrons.

The finite-temperature quantum-mechanical desorption model is integrated with a thermal model to quantitatively predict the sintering rate *R*_sinter_ as a function of laser fluence *F* (Supporting Information), enabling a direct comparison with the sintering rates measured by the *in situ* transmission measurement. Figure 3g shows the calculated superlinear relationship characterized by an exponent of approximately *n* ∼ 6.2. The model suggests that the temperature of the nanocrystal film rises to ∼600 K with a laser fluence of 60 mJ/cm^2^ (Figure S10), which plays a critical role in enabling the superlinear sintering rate. Furthermore, the model predicts an increase (by 3–4 times) in the sintering rate by raising the substrate temperature from 25 to 88 °C (Figure 3h). The predicted superlinear relationship (and exponent *n*) and the sintering rate dependence on the substrate temperature is in reasonable agreement with experimental results verifying the thermally assisted hot-electron-driven sintering mechanism. The exponent *n* = 6.7, as measured by the *in situ* transmission measurement, appears to be higher than the *n* = 3.59 value obtained by fitting the line width with laser fluence (Figure 2b). The difference is attributed to variation in the temperature response during laser irradiation (see the Supporting Information).

Laser sintering was employed to fabricate the various nanostructures. Figure 4a shows SEM images of serpentine lines with varying gaps between parallel segments, ranging from ∼216 to ∼40 nm. Figure 4b presents high-magnification views of various end-to-end gaps. Gaps as small as ∼35 nm were demonstrated. The clear separation between lines, even at sub-100-nm scales, underscores the capability of this process to define sharp boundaries. Figure 4c shows the periodic concentric circular antennas.^27,70^ In Figure 4d, a metasurface of single-nanorod meta-atoms and coupled-nanorod meta-atoms consisting of two nanorods was fabricated with spatial uniformity over tens of microns. Plasmonic nanodots are shown in Figure 4d. Figure 4f shows a metasurface consisting of V-shaped phase-shifting antennas, which can generate spiral interference light intensity.^61^ Figure 4g shows a two-dimensional metasurface composed of nanorods with different lengths and widths for full-Stokes imaging.^71^

**Figure 4:**
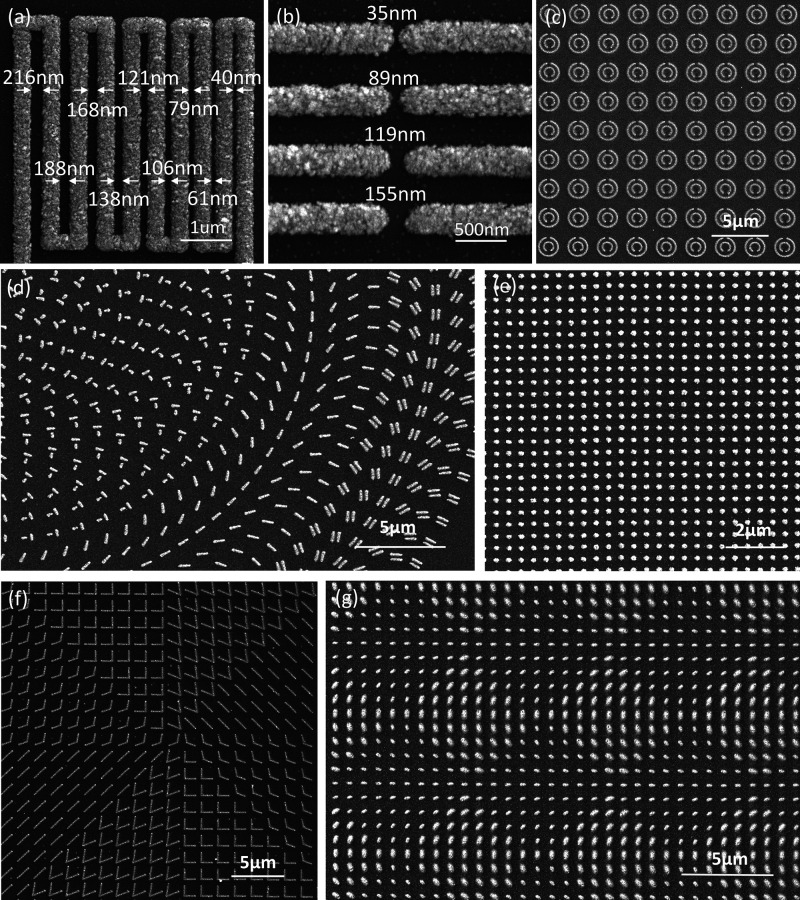
Gaps fabricated between lines and metasurface fabrication. Fabricated gaps between (a) parallel segments and (b) terminals of two sintered lines. Metasurfaces fabricated by the laser sintering process include (c) an array of concentric circular antennas, (d) a metasurface of single-nanorod and coupled-nanorod meta-atoms, (e) an array of plasmonic dots, (f) a phase-shifting metasurface for generating optical vortices, and (g) a phase-shifting metasurface for changing contrast.

To verify the performance of the metasurface fabricated by the laser sintering, a series of bidirectional metasurfaces were fabricated with periodic arrays of gold single nanorods, as shown in Figure 5a. Each gold nanorod undergoes strategic rotation to induce a specific phase shift, thereby forming a spatially varying phase profile across the supercell. The electric field distribution using finite-difference time-domain techniques was simulated (see the Supporting Information). As demonstrated in Figure 5b, the metasurface refracts incident light differently based on whether it is left or right circularly polarized (LCP/RCP). The designed metasurfaces were fabricated, as shown in Figure 5c. As depicted in Figure 5d, by variation of the number of unit cells in a supercell, the refraction angle can be influenced. A higher number of unit cells (*N*) leads to a reduced phase gradient, resulting in smaller refraction angles, consistent with the generalized Snell’s law.^72^ Our results show that a supercell with *N* = 3 exhibits a steeper phase gradient and, thus, a larger refraction angle than one with *N* = 4. Simulated refraction angles are marked with yellow stars in Figure 5d (see the Supporting Information), matching experimental observations. The metasurface maintains efficient light manipulation across a narrow broadband wavelength range from 950 to 1050 nm. Figure 5e illustrates the spectral dependence of the refraction angle, which increases with the wavelength. Figure 5f presents the experimental results of the bidirectional behavior of the metasurface in momentum space. Depending on the circular polarization of the incident light, the beam diffracts into the +1 or −1 order. Collectively, these results underscore the potential of polarization-engineered metasurfaces for compact, tunable photonic devices that exploit phase-gradient control to manipulate the light direction with high fidelity.

**Figure 5:**
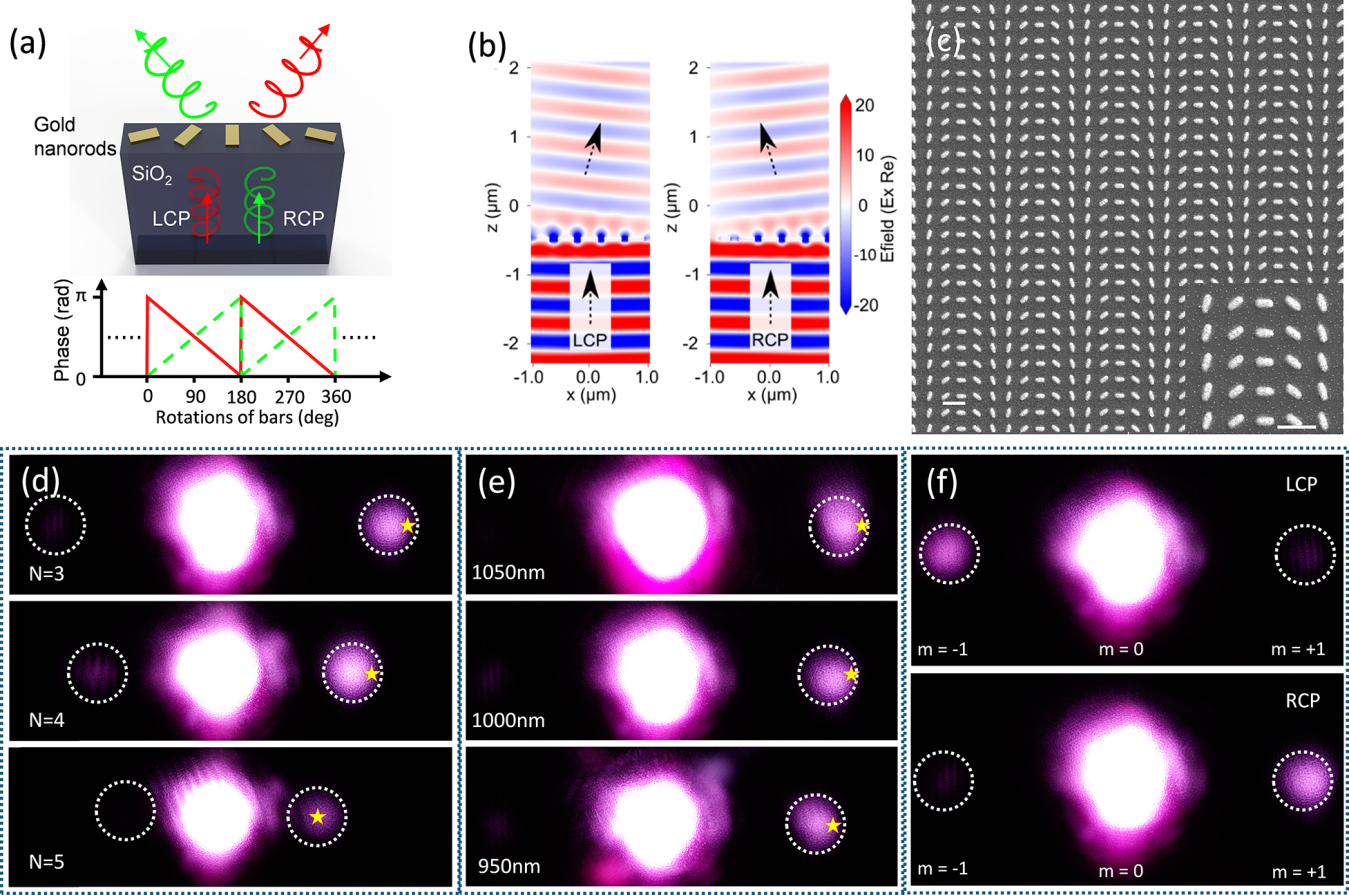
Design, simulation, fabrication, and characterization of the bidirectional metasurface. (a) Schematic of the metasurface supercell consisting of rotated gold nanorods on a glass substrate. (b) Simulated electric field profiles showing bidirectional refraction for LCP and RCP incident light. (c) SEM image of the fabricated metasurface. The nanorod dimensions are 212.5 ± 10.6 nm (mean ± SD) in width and 487.5 ± 9.7 nm in length, with an atomic force microscopy measured surface roughness of Ra = 1.4 nm. The inset shows a magnified view of the supercell design (scale bar is 1 μm). (d) Refraction angle versus supercell configuration (*N* = number of unit cells). (e) Wavelength-dependent refraction behavior of the metasurface. Refraction angles increase with incident wavelength. (f) Experimental momentum-space measurements confirming bidirectional refraction.

In summary, we have demonstrated a laser sintering strategy for the direct writing of plasmonic optical metasurfaces. Metasurfaces, including phase-shifting antennas and polarization-sensitive plasmonic devices, are fabricated and demonstrated. Looking forward, the technology is well positioned to achieve enhanced scalability and versatility through advancements in parallel direct writing, broader material compatibility, and three-dimensional-structuring capabilities. The demonstrated ultralow-power (∼a few microwatts) processing, which imposes minimal thermal burden, is instrumental in enabling parallel direct writing with massive beamlets and *layer-by-layer* three-dimensional nanostructuring. The use of aliphatic ligands, without reliance on specialized photosensitive ligands, suggests their applicability to a broader range of materials.

## Supplementary Material



## Notes

Additional information on the LDW scheme, laser-driven photochemical reaction model derivation, transmission measurement, Raman spectra, quantum-mechanical hot-electron-driven ligand desorption model, metasurface simulation and measurement, and atomic force microscopy characterizations (PDF)
