# Supplementary material for: Direct Writing of Nanostructured Metasurfaces by Hot-Electron-Driven Laser Sintering
Source: Nano Lett. 2025 Oct 6;25(41):15098–106. doi: 10.1021/acs.nanolett.5c04174 (PMC12532292; doi:10.1021/acs.nanolett.5c04174)
Supplement: Supplementary file 1 [file nl5c04174_si_001.pdf]

Supporting Information

Direct Writing of Nanostructured Metasurfaces by Hot-electron-driven Laser Sintering

*Kai Chang, Kai Wei, Kaushik Kudtarkar, Cagatay Yelkarasi, Ali Erdemir, Shoufeng Lan, M. Cynthia Hipwell, Heng Pan**

J. Mike Walker '66 Department of Mechanical Engineering, Texas A&M University, College Station, Texas 77845, USA

*Corresponding author: hpan@tamu.edu

A. Laser direct writing scheme

A cover glass was initially sputter-coated with a 0.1nm (or 0.2 nm) platinum layer as adhesion layer. Subsequently, Oleylamine-capped gold nanocrystal ink (3-5 nm size in Xylene, 25wt%, UTDAu25X, UTDots) was spin-coated onto the glass at 1000 RPM for 1 minute to form a uniform film of ~215 nm thickness. The cover glass was mounted on a 3-axis piezo stage (Nano-LP300, MCL) for precision positioning and movement control. A nanosecond 355 nm laser spot (AO-355A, CNI Laser) with ~20ns pulse duration and 100kHz repetition rate was expanded by a beam expender and then focused through the backside of the cover glass onto the nanocrystal film with a 40x oil immersion objective lens (N.A. = 1.35, UApo/340, Olympus) to initiate the sintering process. A shutter was placed in the beam path to enable precise on-off control of the laser exposure on the film. A customized LabVIEW code was developed to synchronize the operation of the shutter and the piezo stage, allowing for the fabrication of various metasurface patterns. Following laser processing, the substrate was immersed in xylene for 15 minutes to remove the unsintered nanocrystals. The sample was then air-dried for 30 minutes to evaporate any residual xylene solvent.

Alternatively, the laser can be focused onto the nanocrystal film from the front side using a 50× objective lens, enabling direct writing of metasurfaces on opaque substrates such as Si. Based on the estimated laser penetration depth of ~60-70nm in the spin-coated nanocrystal films, the front side configuration requires spin-coating of thinner films. To obtain thinner films, spinning speed is increased to 6000 RPM to obtain films of ~60-70nm thickness. Furthermore, an adhesion layer (e.g., Polyimide) can be introduced to improve film adhesion in the front-side irradiation configuration.

The laser wavelength was selected based on photon energy needed to generate energetic hot electrons to facilitate ligand desorption. The energy level formed by the ligand-metal complex is ~4.0eV above the Fermi level (as shown by DFT simulation in the Section E). A photon energy of 3.5 eV was selected in the

present study, being close to 4.0 eV. The nanosecond laser pulse duration was chosen in order to limit the thermal diffusion and maintain high resolution of the laser direct-writing scheme.

B. Laser-driven photochemical reaction model

It is assumed the reaction rate coefficient of the laser-induced hot-electron driven reaction or desorption can be modeled with a power law:

$$k = aF^n \tag{1}$$

where k is the reaction rate coefficient, F is the laser fluence, and a is a constant, the hot-electron driven (laser induced) ligand desorption rate $\partial\alpha / \partial t$ which determines the un-desorbed ligand concentration can be calculated with the following rate equation,

$$\frac{\partial\alpha}{\partial t} = -k\alpha = -aF^n\alpha \tag{2}$$

$$\alpha = \alpha_0 \exp(-aF^n t) \tag{3}$$

where α is the un-desorbed ligand concentration, α_0 is the initial ligand concentration before laser exposure and t is the laser exposure time. Considering a nanosecond laser with repetition rate f_{rep} (100k Hz) and pulse duration t_p (20 ns), the exposure time t can be calculated as, $t = t_{on} f_{rep} t_p$ wherein t_{on} denotes the laser on-time. When the un-desorbed ligand concentration reduces to a threshold level, the nanoparticle starts to fuse together and sintering occurs.

Due to the Gaussian distribution of the beam on the sample, the local fluence at location x relative to the center of the beam can be described as:

$$F(x) = F_0 \exp\left(\frac{-2x^2}{\omega_0^2}\right) \tag{4}$$

where ω_0 is the radius of the laser spot, F_0 is the laser fluence at the spot center $F_0 = \frac{2e^2}{e^2-1}F \approx 2.3F$, F is the laser fluence $F = \frac{E}{\pi\omega_0^2}$, and E is the laser pulse energy. Then the local ligand concentration after time t within the laser spot is described as the following equation.

$$\alpha = \alpha_0 \exp\left\{-a\left[2.3F \exp\left(\frac{-2x^2}{\omega_0^2}\right)\right]^n t\right\} \tag{5}$$

When the ligand concentration is reduced to the threshold $\alpha_{\text{threshold}}$, sintering occurs,

$$\alpha = \alpha_{threshold} \tag{6}$$

Laser exposure time can be estimated by assuming $t_{on} = \frac{2\omega_0}{v}$

$$t = \frac{2\omega_0}{v} f_{rep} t_p \tag{7}$$

where v is the laser scanning speed, hence the sintered linewidth d can be calculated as follows

$$d = 2x_{threshold} = \omega_0 \sqrt{\frac{2}{n} \ln\left[\frac{2a\omega_0 f_{rep} t_p (2.3F)^n}{\mathrm{v} \ln\left(\frac{\alpha_0}{\alpha_{threshold}}\right)}\right]} \tag{8}$$

where $x_{\text{threshold}}$ is the location where $\alpha=\alpha_{\text{threshold}}$. Equation S8 can be written in the following form

$$d = \omega_0 \sqrt{\frac{2}{n} \ln\left(\frac{C_t \omega_0 (2.3F)^n}{v}\right)} \tag{9}$$

or

$$d = \omega_0 \sqrt{2\ln(2.3\mathrm{F}) + \frac{2}{n}\ln\left[\frac{C_t\omega_0}{\mathrm{v}}\right]}$$

where $C_t = 2af_{rep}t_p/\ln(\frac{\alpha_0}{\alpha_{threshold}})$ is a parameter determined by the sintering threshold.

Equation S9 can be used to fit the experimentally obtained relationship between laser fluence F, laser scanning speed, and linewidth d and obtain the fitted values for ω_0, C_t and n.

By re-arranging Equation S9:

$$\frac{d^2}{2} = -\frac{\omega_0^2}{n}\ln(v) + \omega_0^2\ln(F) + \omega_0^2\ln(2.3) + \frac{\omega_0^2}{n}\ln(C_t\omega_0) \tag{10}$$

and

$$\frac{d^2}{2} = -\frac{\omega_0^2}{n}\ln(v) + \omega_0^2\ln(E) - \omega_0^2\ln(\pi\omega_0^2) + \omega_0^2\ln(2.3) + \frac{\omega_0^2}{n}\ln(C_t\omega_0) \tag{11}$$

Equation S11 can be written as

$$\frac{d^2}{2} = -\frac{{\omega_0}^2}{n}\ln(v) + {\omega_0}^2\ln(E) + C \tag{12}$$

with $C = -\omega_0^2\ln(\pi\omega_0^2) + \omega_0^2\ln(2.3) + \frac{\omega_0^2}{n}\ln(C_t\omega_0)$

The spot size $2\omega_0$, n and C can be obtained through a regression analysis, with laser pulse energy E and scanning speed v as independent variables and d as dependent variable, by fitting Equation S12. The obtained $2\omega_0$=442nm, n=3.59, and C = 9.64×10^{-13} m^2. C_t is found to be 8.40×10^{-11} $(J/m^2)^{-3.59}s^{-1}$, and a ~ 1.45×10^{-8} $(J/m^2)^{-3.59}s^{-1}$.

C. Transmission measurement and sintering rate characterization

To investigate the kinetics of the laser-induced hot electron driven sintering process, an *in-situ* transmission measurement system was developed, as shown in Figure S1. Gold nanocrystal ink was spin-coated onto a glass substrate to form a uniform thin film with thickness ~200nm. A laser beam (355 nm) was focused onto the film through a 40x oil immersion objective lens (N.A. = 1.35, Uapo/340, Olympus) to induce the sintering process (Figure S1a). Simultaneously, an IR beam (1064 nm) was coaxially aligned with the 355 nm laser beam via a pellicle beam splitter. The 1064nm beam spot size was controlled to be smaller than that of the 355 nm beam. A photodiode (DET100A, Thorlabs), sensitive to wavelengths in the 400-1100

nm range, was placed directly beneath the sample to convert the transmitted 1064nm radiation into an electrical signal. This signal was monitored using an oscilloscope to capture the *in-situ* IR transmission. To ensure efficient signal collection, an achromatic doublet lens with a 30 mm focal length and anti-reflective coating (ARC) for wavelengths between 650-1000 nm was positioned between the sample and the photodiode, collimating the transmitted 1064nm beam and ensuring optimal detection by the photodiode. To prevent alterations in the nanocrystals, the power of the probing infrared (IR) radiation was kept sufficiently low.

As sintering progresses, the fused nanocrystals reduce the transmission of the probing 1064 nm radiation, making the transmitted signal a useful metric for assessing the degree of sintering. Figure S2 shows representative transmission signal changes at different laser fluences, highlighting the temporal evolution during nanoparticle sintering. To quantify the transient sintering process, the sintering time τ_{sinter} is defined as the duration required for the transmission signal to decrease to 90% of the initial transmission (Figure S2). The sintering rate R_{sinter} is defined as the reciprocal of the sintering time τ_{sinter}.

To further investigate the role of temperature in the sintering kinetics, an *in-situ* heater was incorporated to alter the temperature of the substrate T_{sub}. The substrate was heated to approximately 88 °C (T_{sub} = 88°C) using a flexible heater controlled by a temperature controller (Figure S1b). A 20X objective lens was used to focus the processing laser (355nm) and probing laser (1064nm). Sintering rates were measured at the elevated temperature and room temperatures.

D. Residual ligands in the laser-sintered films

To investigate the residual ligands in the laser-sintered films, Raman spectra were collected from laser-sintered films subjected to additional thermal sintering at 200 °C and 350 °C (Figure S3a). The emergence of D and G bands after thermal sintering at 200 °C of laser-sintered films indicates the presence of residual ligands in the laser-sintered film, which undergo thermal decomposition during the additional thermal sintering process. Further thermal sintering at 350 °C leads to a reduction in the D and G bands, suggesting

additional removal of residual carbonaceous materials. A similar evolution of ligands is observed in the thermally sintered films (Figure S3b). Thermal sintering of as-deposited films at 200 °C reveals the thermal decomposition of ligands, as evidenced by the strong D and G bands, albeit the intensities are significantly higher than those in thermal sintering of laser-sintered films (Figure S3a). The reduced intensity reflects prior ligand evolution due to laser desorption and the resulting decrease in the ligand amount prior to the additional thermal sintering. Subsequent sintering at 350 °C further reduces the residual carbon content, reaching levels comparable to those in the laser-sintered films subjected to the same 350 °C thermal treatment.

To further validate the photoexcited hot electron driven mechanism of the sintering process, a laser operating at 1064 nm with identical pulse duration and repetition rate was used for comparison. The IR laser (1064 nm wavelength) was focused with a 20x objective lens to sinter the spin-coated nanocrystal films and laser fluence was increased until laser processing was observed. At laser fluence at ~188 mJ/cm^2, noticeable laser processing can be observed. The optical images and Raman spectra of the processed films at 188 mJ/cm^2 and 377 mJ/cm^2 fluences are shown in Figure S4. Both fluences result in prominent D and G peaks in Raman spectra and no highly reflective films can be obtained, which indicates the IR laser (1064 nm) tends to drive direct photothermal carbonization of the ligands rather than the hot-electron driven ligand desorption as observed by the 355 nm laser.

E. The quantum mechanical model for the thermally assisted hot electron driven sintering

The quantum mechanical model consists of the following steps. First, a finite temperature quantum mechanical ligand desorption model is developed to predict the probability of ligand desorption per photoexcited hot electron. Second, a model is developed for hot electron flux in a nanocrystal exposed to laser irradiation. Third, based on 1 and 2, reaction rate coefficient can be calculated. Then a thermal model is developed to estimate the temperature rise during laser irradiation which can be combined with step 1-3

to predict the relationship between laser fluence and reaction rate coefficient. Finally, the relationship between reaction rate coefficient and sintering rate is established.

The ligand desorption model

The theoretical framework for describing the nonadiabatic dynamics resulting from the interaction of a hot electron with an adsorbate (or ligand) is based on the concept of potential energy surfaces (PES). Under the Born-Oppenheimer approximation, electrons are assumed to remain in their ground state. However, when an initially unoccupied electronic resonance becomes occupied, a new excited-state PES emerges. This excited-state surface typically has its minimum at a different position compared to the ground-state PES, resulting in a force being exerted on the adsorbate.

To determine ground state and excited state PESs, Density Functional Theory (DFT) calculations were performed using the GPAW code, a real-space grid-based DFT implementations with the projector-augmented wave method. The exchange-correlation interaction was approximated using the PBE function. To model the adsorbate (ligand)-metal complex, a methylamine molecule was adsorbed to the top site of the Au (111) surface as shown in Figure S5a. The Au (111) surface was modeled by a 3×3×2 slab with 15 Å of vacuum space using 13 irreducible k-points and a grid spacing of 0.2 Å. Spin-polarization was included in all the calculations. A finite temperature Fermi function ($k_BT = 0.1$ eV) was utilized to facilitate the SCF convergence by smearing the band occupation.

The energy levels HOMO and LUMO of the methylamine molecule and their hybridization with gold atoms are most relevant in understanding the adsorption and desorption behaviors. The total density of states (DOS) of methylamine-gold complex is shown in Figure S5b along with the projected density of states (PDOS) of HOMO and LUMO of the methylamine molecule.

The potential energy surfaces (PES) of both the ground and excited states of methylamine on Au(111) were obtained by calculating the free energy as a function of the molecule–metal separation, specifically the N-

Au distance. To model the excited state, the linear expansion Δ Self-Consistent Field (ΔSCF) method was employed. In this approach, an electron excitation from the Fermi level of the metal to the LUMO of the methylamine molecule was explicitly considered. Figure S5c depicts the calculated potential energy surfaces (PES) of the ground and excited states. The equilibrium position of the ground state is found to be 2.465 Å, and the resonance energy of the methylamine LUMO orbital is calculated to be approximately 4.1 eV.

After obtaining the PES of the ground state and excited state, the absorbate (ligand) desorption is modeled as one-dimensional desorption problem driven by hot electrons[1]. The one-dimensional adsorbate desorption model can be constructed by considering the vibrational energy of the adsorbate relative to the metal substrate. In this model, the dynamics are governed by a potential energy surface that describes the interaction between the adsorbate and substrate, allowing for the calculation of desorption probabilities as a function of vibrational excitation and hot electron excitations. A general nonadiabatic Newns-Anderson-type model is applied to the one-dimensional desorption problem with model parameters. The parameters in desorption model are the width of the resonance Γ, the frequencies of the normal mode ω, the resonance energy ε_a and the coupling coefficient λ. The resonance was modeled as a Lorentzian function of the width Γ of 1.5 eV and ε_a = 4.1 eV. The vibrational frequency of the N-Au bond ω was calculated to be 16.4 meV based on the ground state PES of the methylamine-gold system described in Figure S5c. The force imposed on the ligand due to the formation of excitation was evaluated by taking the derivative of the excited PES at the equilibrium position of the ground state PES and the coupling coefficient λ is found to be 20.8 meV.

To model the desorption rate, it is essential to compute the probability for the ligand to be excited from an initial vibrational state m to a final state n via inelastic scattering with a single electron of incident energy ε_i. The probability $P(m \rightarrow n)$ is evaluated as:

$$\mathrm{P(m \rightarrow n)} = \Gamma^2 e^{-2g(1+2m)} \left[\frac{g^{n-m}(1+m)^{n-m}}{(n-m)!} F(n-m,0) + \frac{g^{n-m+1}(1+m)^{n-m+1} gm}{(n-m+1)!} F(n-m+1,1) + \frac{g^{n-m+2}(1+m)^{n-m+2}(gm)^2}{(n-m+1)!\,2!} F(n-m+2,2) \right]$$

(13)

wherein function F is calculated using the following:

$$F(m_1, m_2) = \left| \sum_{i=0}^{m_1} \sum_{j=0}^{m_2} (-1)^{i+j} \binom{m_1}{i} \binom{m_2}{j} \sum_{k=0}^{\infty} \sum_{l=0}^{\infty} \frac{g^{k+l}(1+m)^k n^l}{k!\,l!} \frac{1}{\varepsilon_i - \varepsilon_a - (i-j+k-l-g)\hbar\omega + i\Gamma/2} \right|^2$$

(14)

wherein $g = (\frac{\lambda}{\hbar\omega})^2$ corresponds to the dimensionless coupling constant, ε_i is the incident electron energy, ε_a is the resonance energy, Γ is the width of the resonance as described previously, which is related to the lifetime of the excited electron.

Using the function $P(m \rightarrow n)$ and a given initial distribution of vibration states $p(m)$, one can calculate the probability of the absorbate (ligand) being in the nth vibrational state after an inelastic electron scattering:

$$Q(n) = \sum_{m=0}^{\infty} p(m) P(m \rightarrow n)$$

(15)

The initial distribution of vibrational states of the absorbate (ligand) depends on the temperature of the methylamine-gold system. To consider the temperature effect, a finite temperature electron scattering approach[2] is taken by considering the Bose–Einstein distribution for p(m):

$$p(m) = \frac{1}{e^{\left(m+\frac{1}{2}\right)\hbar\omega/k_BT} - 1}$$

(16)

where T is the temperature of the methylamine-gold system.

The probability of the absorbate (ligand) being in the nth state can then be used to calculate the desorption probability induced by a single incident electron with energy ε_i,

$$R(\varepsilon_i) = \sum_{n=n_R}^{\infty} Q(n, \varepsilon_i)$$

(17)

where n_R satisfies the desorption condition $(n_R+1/2)\,\hbar\omega \geq E_D$ and E_D is the desorption energy. It is assumed desorption occurs immediately if the vibrational energy reaches the desorption energy E_D (0.5 eV).

With R, the desorption probability per photoexcited hot electron per adsorption site can be written as:

$$Desorption\ probability\ per\ photoexcited\ hot\ electron = \int_{E_F}^{\infty} R(\varepsilon, T) f(\varepsilon)\, d\varepsilon$$

(18)

where $f(\varepsilon)$ is the energy distribution of photoexcited hot electron. It is noted that $\int_{E_F}^{\infty} f(\varepsilon) d\varepsilon = 1$. The calculated desorption probability per photoexcited hot electron using Equ.S6 is shown in Figure S6.

The hot electron flux and energy distribution

To obtain $f(\varepsilon)$, the generation of hot electrons in nanocrystals and their energy distribution upon laser irradiation can be calculated by real-time time dependent density functional theory (RT-TDDFT) [3,4] using the GPAW code. A methylamine in the (111) on-top sites at 2.7 Å from the Au_{201} nanocrystal was

considered (Figure S7a). A Gaussian pulse of frequency 3.5 eV (355nm) with a 10 femtoseconds (fs) pulse width (5fs FWHM) and peak electric field (5.13×10^3 V/cm) is used to excite the nanocrystal (Figure S7b). The peak electric field corresponds to a peak intensity of 3.51×10^4 W/cm^2 and the specified pulse shape corresponds to an averaged intensity of 1.75×10^4 W/cm^2 over 10 fs pulse duration.

The hot electron generated by the pulse in the nanocrystal can be calculated by following the procedure described in the references[3,4]. The number of hot electrons is plotted in Figure S7c. It can be seen that with the averaged intensity of 1.75×10^4 W/cm^2 over 10 fs pulse duration, the total number of hot electrons generated per nanocrystal is 2.78×10^{-7} as evaluated at ~30 fs (arrow in the Figure S7b. Based on this, the hot electron flux per nanocrystal is $J_0 = 2.78\times10^7$ per second ($2.78\times10^{-7}/10\times10^{-15}$s) at 1.75×10^4 W/cm^2 laser intensity, and the hot electron flux per nanocrystal J at intensity I can be calculated as,

$$J(I) = \frac{I}{1.75 \times 10^4} J_0 = 2.78 \times 10^7 \frac{I}{1.75 \times 10^4} \tag{19}$$

Since the flux of hot electron in the nanocrystal can be written as,

$$J(I) = \frac{\sigma I}{\hbar v} \tag{20}$$

where σ is absorption cross-sectional area of the nanocrystal, and $\hbar v$ is the photon energy. Based on Equation S19 and S20, it can be determined that $\sigma = 8.89\times10^{-16}$ cm^2 for the nanocrystal (Au_{201}).

The intensity I can be evaluated by F/t_p (W/cm^2) with laser fluence F and pulse duration t_p:

$$I = F/t_p \tag{21}$$

To obtain the energy distribution of the hot electron above Fermi level, the number of electrons and holes are evaluated at 30 fs (labeled as the arrow in Figure S7c) after the laser pulse. The number of electrons and holes are normalized by the total number of carriers to obtain the energy distribution $f(\varepsilon)$ of hot electrons and holes per photo-excited hot electron, as shown in Figure S8. Hot electron with energy up to ~3.5 eV can be formed above the Fermi level.

Prediction of reaction rate coefficient

Given the flux of hot electrons J per nanocrystal, the reaction rate coefficient k of ligand desorption rate per adsorption site can be calculated by,

$$k(T,I) = \frac{J}{\#}\int_{E_F}^{\infty} R(\varepsilon,T)f(\varepsilon)d\varepsilon = \frac{\sigma I}{\hbar v}\frac{1}{\#}\int_{E_F}^{\infty} R(\varepsilon,T)f(\varepsilon)d\varepsilon = \frac{F}{t_p}\frac{\sigma}{\hbar v}\frac{1}{\#}\int_{E_F}^{\infty} R(\varepsilon,T)f(\varepsilon)d\varepsilon \tag{22}$$

where J represents the flux of hot electrons depending on excitation level or laser intensity which is calculated as F/t_p where F is laser fluence and t_p is laser pulse duration, # is the number of adsorption sites per nanocrystal. It is assumed that there are ~50 surface adsorption sites (# = 50).

The thermal model

Since the temperature increases during the laser pulse and the desorption reaction coefficient k is temperature-dependent, it is necessary to estimate the temperature profile during pulsed laser heating. It can be shown that the peak temperature T_{peak} during the pulsed laser heating of nanocrystal film on the substrate can be calculated as,

$$T_{peak} = \frac{I\alpha}{\rho C h G} + T_{sub}, \text{ with } I = \frac{F}{t_p} \tag{23}$$

where G is cooling rate (K/s) due to heat dissipation to the substrate, C is specific heat of the nanocrystal film (estimated to be 126 J/kg/K), ρ is the density of the nanocrystal film (estimated to be 19300 kg/m^3), h

is the nanocrystal film thickness (estimated to be 200nm), α is the absorptivity (estimated to be 0.7), and T_{sub} is the substrate temperature. The cooling rate G can be estimated to be 1.3×10^{8} K/s based on nanosecond cooling time[5] τ_t = 7.2ns for nanocrystals to dissipate heat to substrate. Based on this thermal model, the temperature can be related to laser fluence as shown in Figure S9.

By substituting T in the reaction rate coefficient $k(T,F,t_p)$ with T_{peak}, the reaction rate coefficient k can be written as a function of F and t_p,

$$k(F,t_p) = \frac{\sigma F}{\hbar v t_p}\frac{1}{\#}\int_{E_F}^{\infty} R\left(\varepsilon, \frac{F\alpha}{t_p \rho C h G} + T_{sub}\right) f(\varepsilon)\, d\varepsilon \tag{24}$$

The relationship between reaction rate coefficient and sintering rate

The kinetics of hot-electron driven nanocrystal sintering can be understood by a reaction model and the reaction rate coefficient k derived previously. The hot-electron driven (laser induced) ligand desorption rate $\partial\alpha / \partial t$ which determines the un-desorbed ligand concentration can be calculated with the following rate equation,

$$\frac{\partial\alpha}{\partial t} = -k\alpha$$

and

$$\alpha = \alpha_0 \exp(-kt)$$

wherein α is the un-desorbed ligand concentration, α_0 is the initial ligand concentration (before laser irraidation), t is the laser exposure time and k is the reaction rate coefficient. Considering a nanosecond laser with repetition rate f_{rep} (100k Hz) and pulse duration t_p (20 ns), the exposure time t can be calculated as,

$$t = t_{on} f_{rep} t_p$$

wherein t_{on} denotes the laser on-time.

According the reaction rate equation, the characteristic exposure time for ligand desorption (or sintering time) is $1/k$. To determine the characteristic laser on-time τ_{sinter} required for sintering, letting $\tau_{sinter} f_{rep} t_p = \frac{1}{k}$, it is found that characteristic laser on-time τ_{sinter} is,

$$\tau_{sinter} = \frac{1}{k f_{rep} t_p}$$

or the sintering rate R_{sinter} is,

$$R_{sinter} = 1/\tau_{sinter} = k(F, t_p) f_{rep} t_p \quad (25)$$

This equation relates the sintering rate R_{sinter} (or measured laser on-time for sintering τ_{sinter}) and reaction rate coefficient k. It allows the prediction of the relationship between R_{sinter} and laser fluence F, which can be compared with experimentally determined relationship.

F. The difference in exponent n

The exponent n = 6.7 as measured by the *in-situ* transmission measurement appears to be higher than the n = 3.59 obtained by fitting the linewidth with laser fluence and laser scanning velocity. The difference is attributed to variation in temperature response during laser irradiation. In the *in-situ* transmission measurement, the sintering rate was evaluated at the center of the laser spot, where the temperature is assumed to be linearly proportional to the laser fluence F_0 (i.e. $T \sim F_0$). In contrast, the linewidth is influenced by the spatial distribution of temperature across the laser spot, which may deviate from the laser profile due to thermal diffusion. The thermal diffusivity of the glass substrate is ~5×10^{-7} m^2 s^{-1} which corresponds ~100 nm heat diffusion length over the period of 20ns laser heating. The thermal diffusion length is comparable to the laser spot radius (ω_0), resulting in a broader temperature distribution than the original laser spot size. Since the sintering rate R_{sinter} (or k) is a function of both local laser fluence and local temperature as

indicated by Equation S22, a broader temperature distribution effectively reduces the power law dependence, leading to a lower exponent n.

To illustrate this, the temperature distribution induced by a pulsed laser with ω_0=100nm and t_p=20ns is computed using a thermal conduction model. As shown in Figure S10, thermal diffusion redistributes absorbed energy outward from the irradiated region, broadening the temperature profile relative to the laser fluence profile. This lateral spreading lowers the peak temperature and suppresses spatial gradients, so the local temperature no longer scales linearly with the local laser fluence, as shown in Figure S10b. Consequently, when the local temperature and fluence are used in Equation S22 to determine the dependence of R_{sinter} on laser fluence F, the result is a nonlinear scaling with a reduced exponent n = 3.04 (Figure S10c), as compared with n = 6.2 in Figure 3g, which is consistent with the experimentally observed difference in n obtained from transmission and linewidth fitting.

G. Phase-shifting metasurface simulation

The simulation was performed using the finite-difference time-domain (FDTD) software Tidy3D. The metasurface was designed with 3 to 5 gold nanorods in each supercell, arranged with equal angular spacing over 180°. The nanorods were incrementally rotated within the supercell, and periodic boundary conditions were applied along the X and Y axes. Perfectly matched layers (PMLs) were implemented along the Z axis to seamlessly absorb outgoing waves. The device, made of gold, is designed to manipulate light with a wavelength of 1000 nm. Each nanorod has 500 nm in length, 225 nm in width, and 79 nm in height, with a pitch of 750 nm in both the X and Y directions.

The electric field of circularly polarized light (λ=1000nm) refracted into the m=+1diffraction order was simulated and its distribution is shown in Figure S11. The refracted angles with different numbers of unit cells are shown in the bottom.

From the electric field distribution result, by changing the number of gold nanorods in a supercell, the phase gradient ratio (dϕ/dx) can be altered methodically, a controlled modulation of optical momentum can be orchestrated. The stratagem here hinges upon the number of nanorods embedded within each supercell as fewer nanorods yield a steeper phase gradient, when N=3, the phase shift is culminating in an angular deflection leading to a refraction of 26 degrees. Conversely, as the number of nanorods increases, the phase shift per unit diminishes, which leads to the angular exuberance of the refracted beam waves.

The refraction in K-space can also be plotted with $K_x = \sin(\theta)$, where K_x is the spatial frequency in x-direction and θ is the refraction angle. The K-space result from different numbers of unit cells with left circularly polarized light is shown in Figure S12. And this result can be used in the comparison of the images got in optical tests.

The K-space result of light with different wavelengths (950nm, 1000nm, 1050nm) were also calculated, the result is shown in Figure S13 to verify the broadband capability of the metasurface.

H. Metasurface optical measurement

The metasurface fabricated was placed on a translation stage between a 10x objective lens (N.A. = 0.28, M Plan Apo, Mitutoyo) for exciting beam and a 40x objective lens (N.A. = 0.75, Plan Flour, Nikon) for collecting the refracted beam. The two objectives were both focused on the metasurface using kinematic mirror mounts (KS1, Thorlabs). A probe beam with varying wavelength from 900 nm to 1050 nm was generated by a femtosecond pulsed laser. Absorptive neutral density (ND) filters were used to attenuate the intensity of the pulsed laser beam. The probing beam passed through a combination of a linear polarizer and a quarter waveplate then incident on the metasurface through the 10x objective lens. The transmitted light was collected by the 40x objective lens. A low numerical aperture (NA) objective serves as the focusing lens, while a high-NA objective is employed to collect light refracted at large angles from the sample. An additional lens is placed at its back focal plane to form an image at the location of a pinhole. This pinhole acts as a spatial filter and restricts the emitted light to ensure the beam is well-aligned on the optical table. A subsequent focusing lens is placed at a distance equal to its focal length from pinhole and guides the light onto the CCD camera. This configuration forms a real-space image of the sample. Alternatively, replacing this lens with one of focal length f/2 produces a Fourier-space image of the refracted beam at the CCD, enabling precise measurement of both the refraction angle and efficiency of light emitted from the sample. The refraction modes and their efficiencies are analyzed by post processing the images of refracted light with a customized MATLAB code.

I. AFM measurement of the thickness of films and laser sintered lines and roughness

A cover glass was first sputter-coated with a 0.2 nm layer of platinum. Oleylamine-capped gold nanoparticle ink was then spin-coated onto the glass with various speeds (1000-6000 RPM) for 1 minute to form a uniform film. A straight line was scratched into the film locally remove the nanocrystal. Atomic force microscopy (AFM) was used to characterize the surface topography around the scratched region, revealing a height difference (film thickness) between the intact film and the bottom of the scratched line. Films with thicknesses of 215–70 nm were obtained by varying the spin-coating speed from 1000 to 6000 RPM.

To determine the laser sintered film thickness, a series of serpentine patterns were fabricated on the spin-coated film using the laser sintering at a constant scanning speed of 0.5 µm/s and varying fluences from ~40 to ~120 mJ/cm². AFM measurements of the resulted patterns indicate the thickness of the sintered lines, as shown in Figure S14a. The dependence of line thickness on laser fluence is presented in Figure S14b.

To determine the roughness, a nanoline was fabricated and scanned along its length using AFM, and the surface roughness was reported as Ra.

J. Electrical conductivity measurement

To measure the conductivity of the laser-sintered structures, nanoelectrodes were fabricated as a series of ~50 µm long nanolines connecting two square contacting pads (Figure S15). Resistance was measured using a probe station coupled to a precision multimeter, while line dimensions were characterized by SEM and AFM. Conductivity was then calculated from the measured resistance and dimensional data.

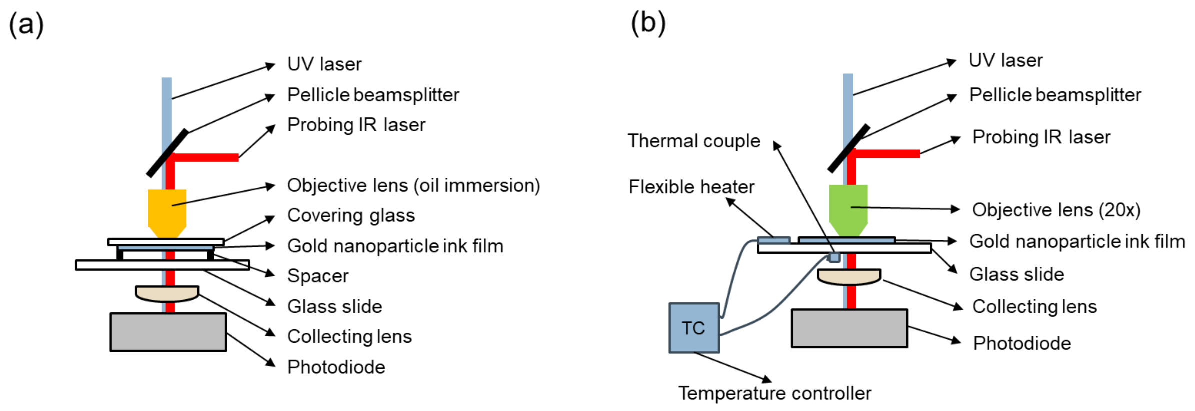

Figure S1. Setup of the transient optical characterizations. (a) the setup of transmission measurement using oil immersion lens in room temperature. (b) the setup of transmission measurements with temperature controlling.

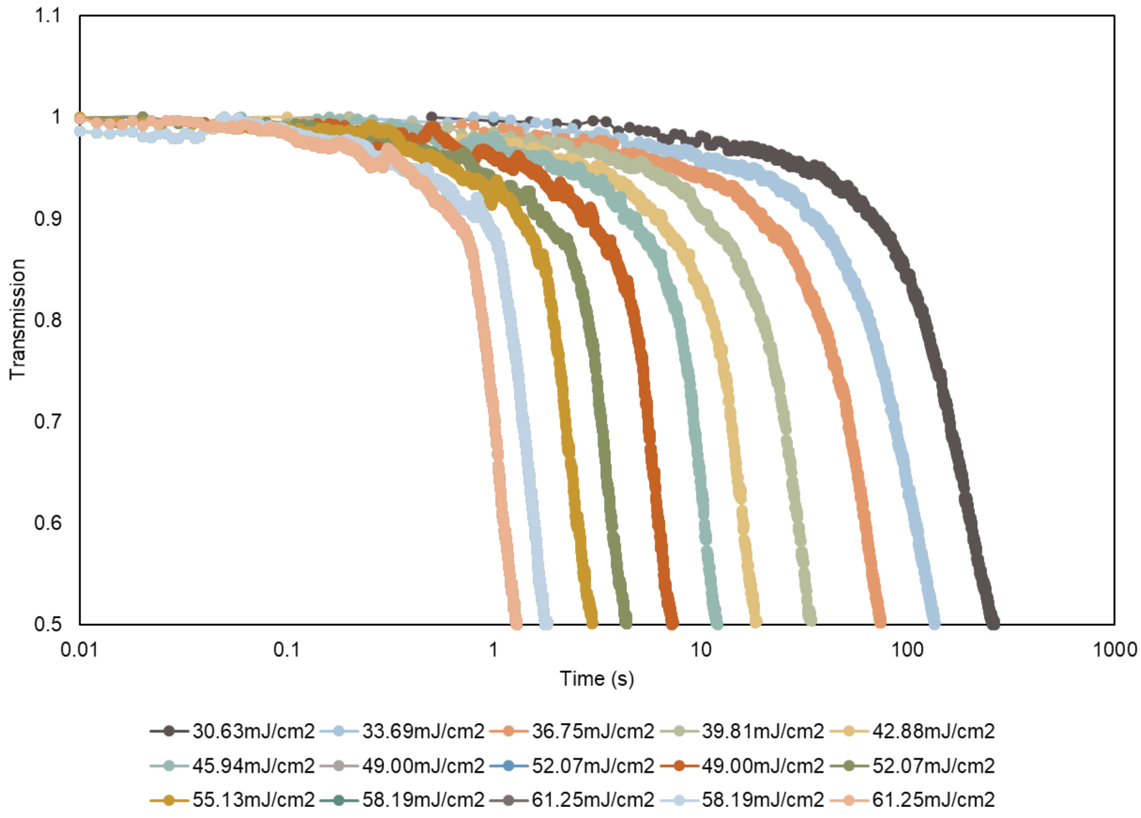

Figure S2. Transient IR transmission signals for various laser fluences during laser sintering.

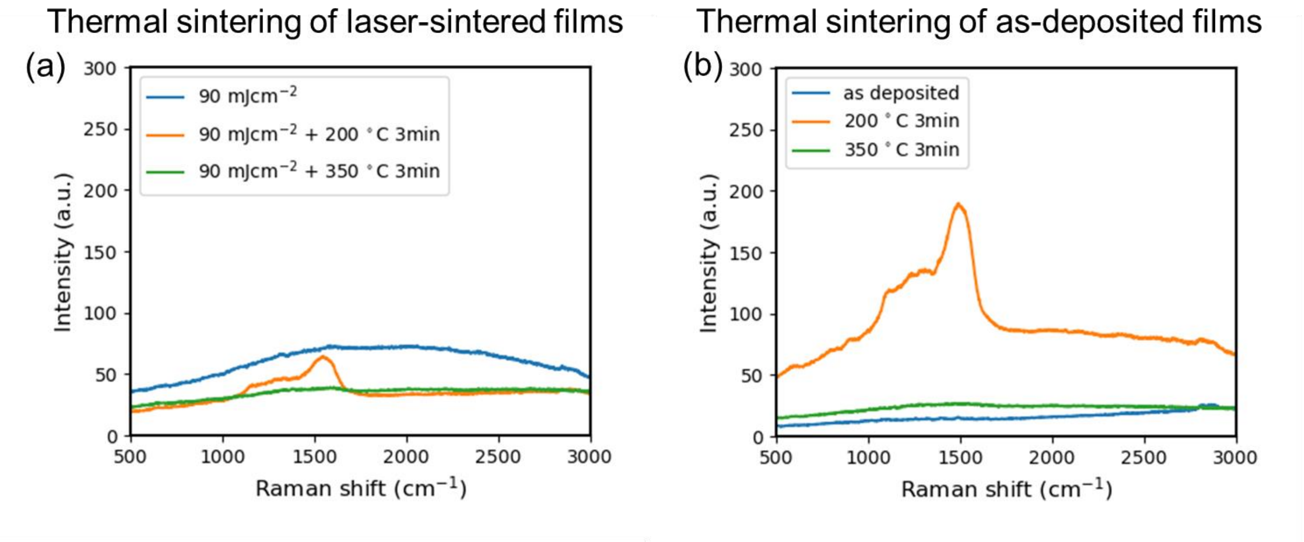

Figure S3. Raman spectra. (a) Thermal sintering of laser-sintered films. (b) Thermal sintering of as-deposited films.

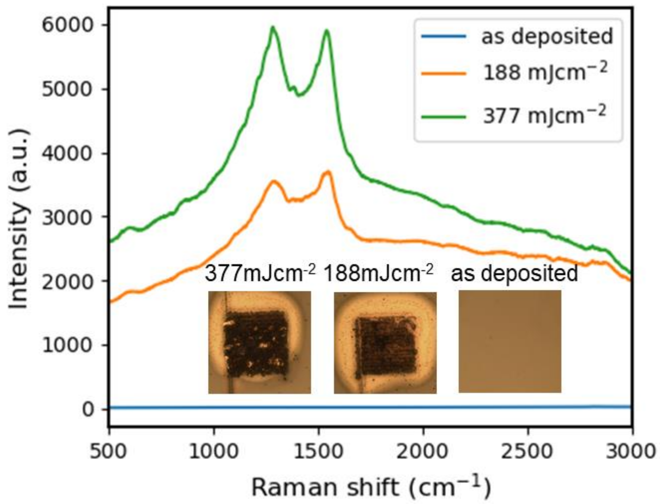

Figure S4. Raman spectra and images of nanocrystal film sintered with 1064 nm laser.

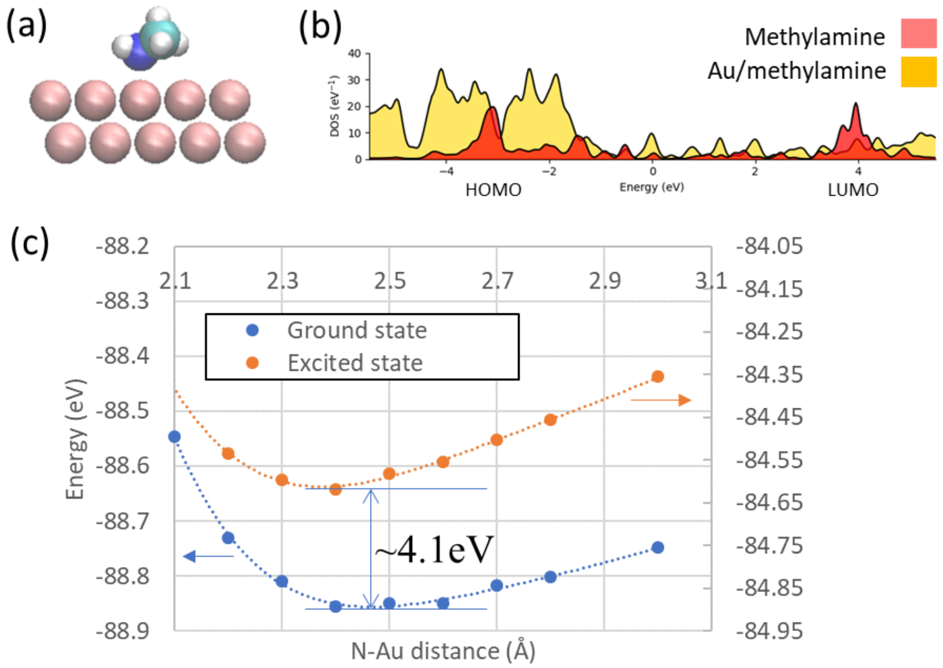

Figure S5. The PES calculation. (a) Methylamine-gold complex in DFT computation. (b) The total DOS of methylamine-gold complex and the projected density of states (PDOS) of HOMO and LUMO of the methylamine molecule (PDOS scaled by 20X in the plot). (c) Calculated ground state and exited state PES.

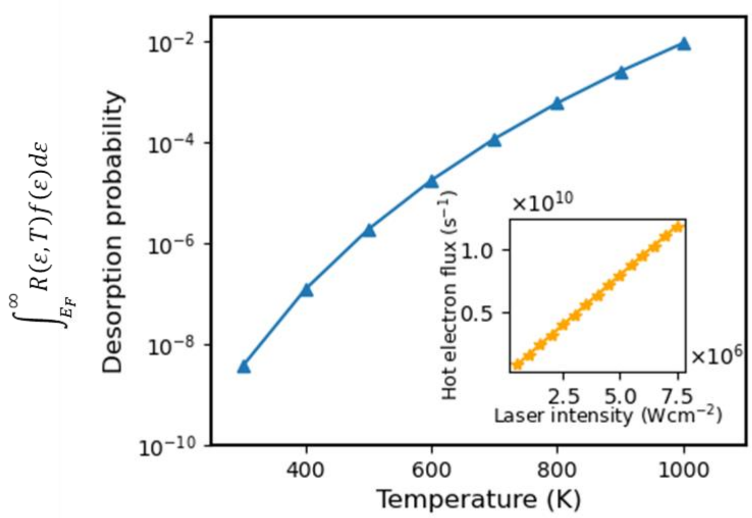

Figure S6. Calculated desorption probability per photo-excited hot electron at various temperatures and hot electron flux in a nanocrystal under various laser intensity excitations (inset).

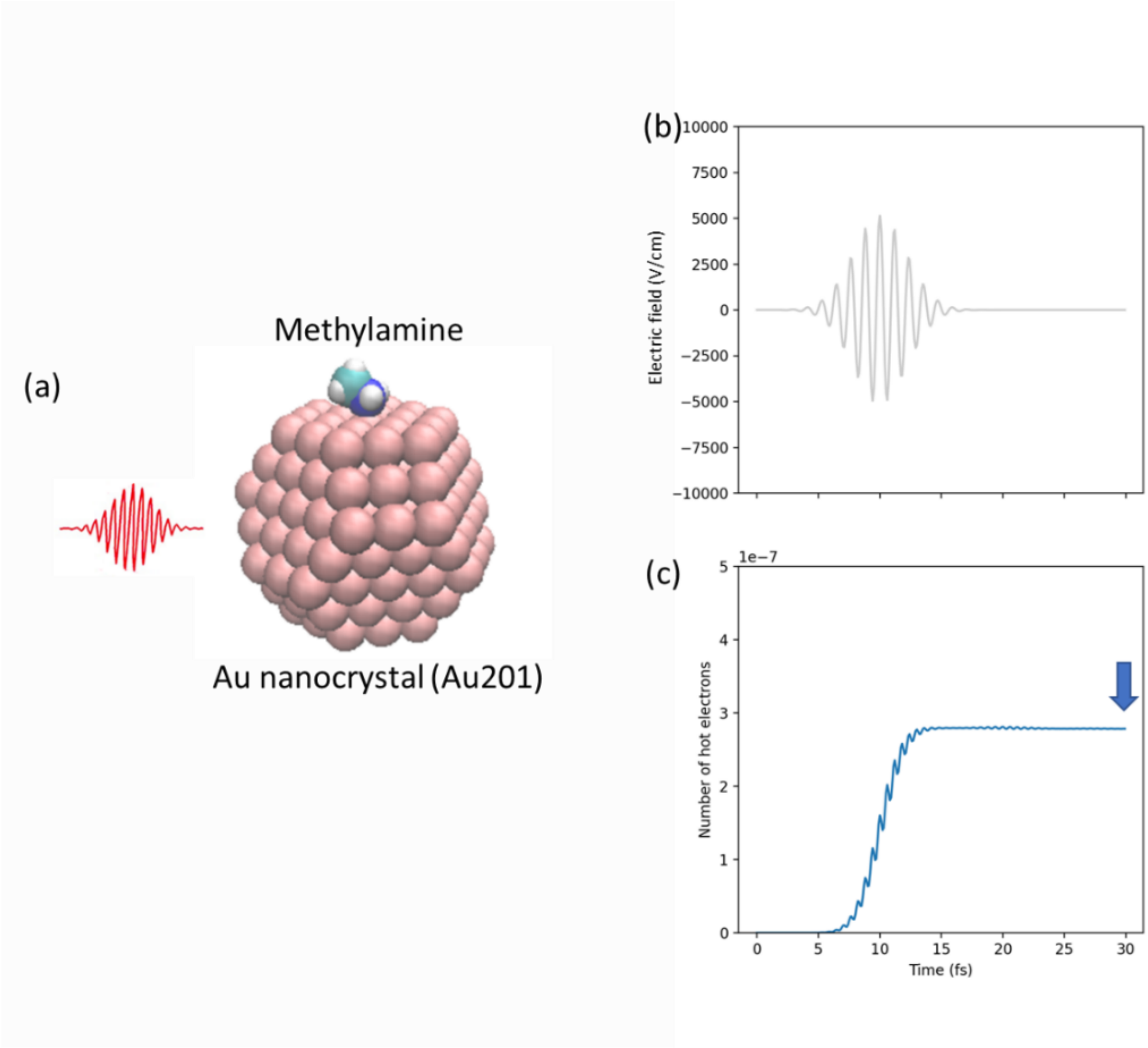

Figure S7. The hot electron distribution and flux calculation by TDDFT. (a) Methylamine-Au201 configuration used in TDDFT calculation. (b) The femtosecond pulse shape used in TDDFT calculation. (c) Calculated hot electrons generated in the nanocrystal as a result of the fs laser pulse excitation.

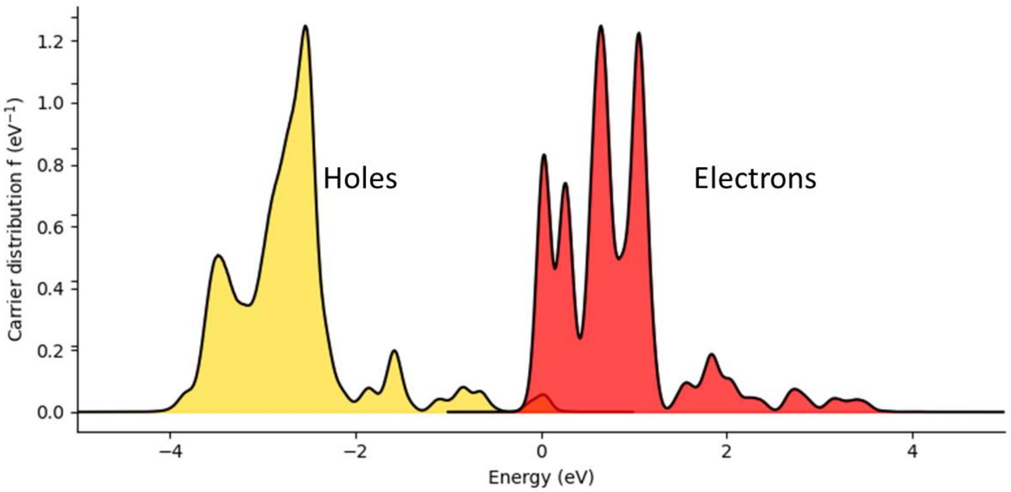

Figure S8. The energy distribution of hot carriers in the Au201 at the end of the pulsed laser (355nm) excitation.

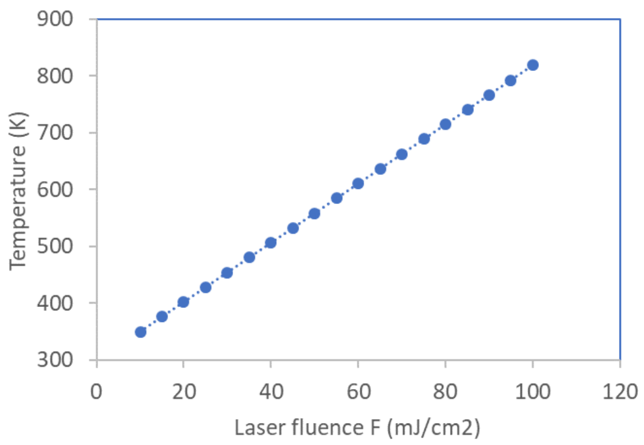

Figure S9. The calculated temperature as a function of laser fluence.

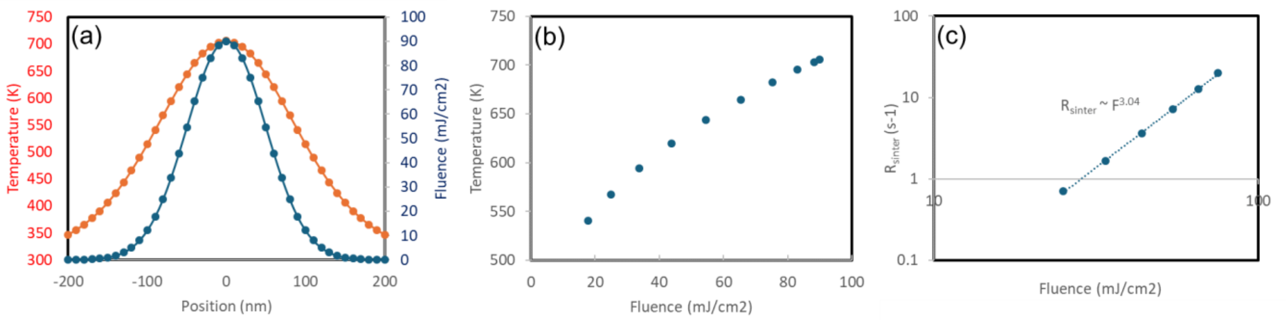

Figure S10. (a) Calculated temperature and laser fluence distribution in the laser focal spot. (b) The dependence of local temperature on local laser fluence. (c) The calculated dependence of sintering rate R_{sinter} on fluence after considering thermal diffusion of a laser focal spot.

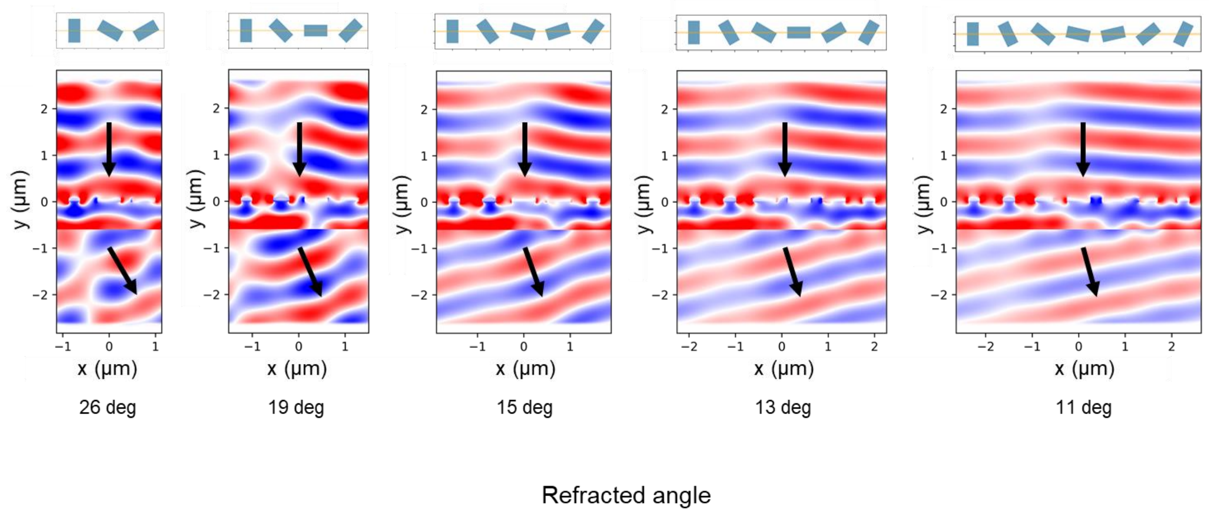

Figure S11. Electric field distributions for circularly polarized light (λ = 1000 nm) refracted into the m=+1 diffraction order by gold nanorods on a glass substrate.

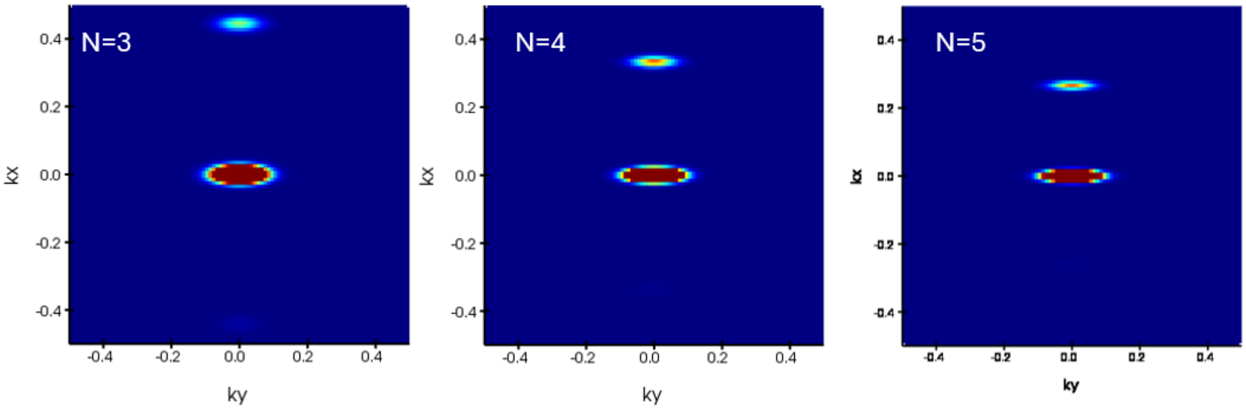

Figure S12. K-space distributions for the left circularly polarized light (λ = 1000 nm) refracted into the m=+1 diffraction order by gold nanorods on a glass substrate.

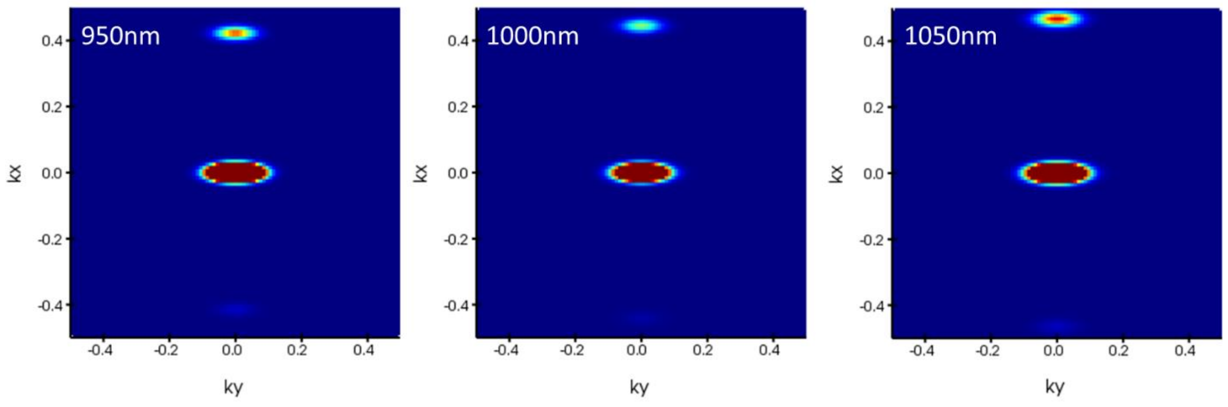

Figure S13. K-space distributions of the left circularly polarized light with different wavelengths refracted into the m=+1 diffraction order by gold nanorods (N=3) on a glass substrate.

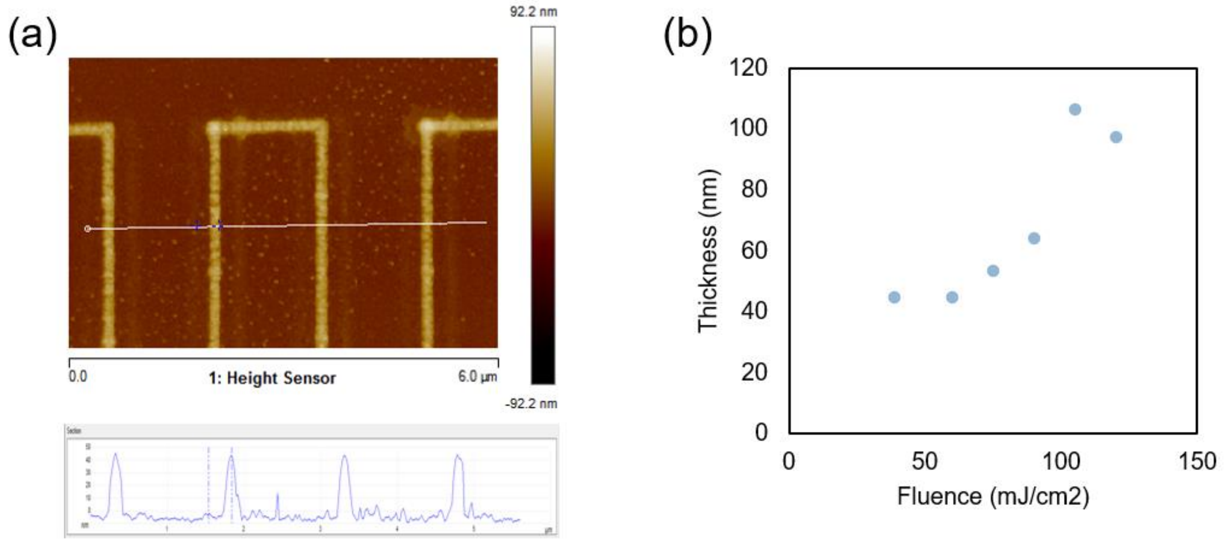

Figure S14. AFM result of sintered patterns. (a) AFM image showing the thickness at different positions along the serpentine line; the bottom panel presents the quantified topography along the section line. (b) Thickness measurements of serpentine lines scanned at different laser fluences with a scan speed of 0.5 µm/s.

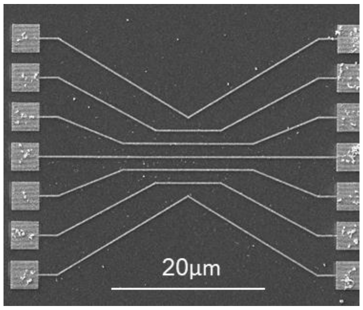

Figure S15. SEM image of nanoelectrodes fabricated for electrical conductivity measurement.

References

(1) Olsen, T.; Gavnholt, J.; Schiotz, J. Hot-electron-mediated desorption rates calculated from excited-state potential energy surfaces. *Phys Rev B* **2009**, *79* (3).

(2) Christopher, P.; Xin, H. L.; Marimuthu, A.; Linic, S. Singular characteristics and unique chemical bond activation mechanisms of photocatalytic reactions on plasmonic nanostructures. *Nat Mater* **2012**, *11* (12), 1044-1050.

(3) Fojt, J.; Rossi, T. P.; Kuisma, M.; Erhart, P. Hot-Carrier Transfer across a Nanoparticle-Molecule Junction: The Importance of Orbital Hybridization and Level Alignment. *Nano Lett* **2022**.

(4) Rossi, T. P.; Erhart, P.; Kuisma, M. Hot-Carrier Generation in Plasmonic Nanoparticles: The Importance of Atomic Structure. *Acs Nano* **2020**, *14* (8), 9963-9971.

(5) Guzelturk, B.; Utterback, J. K.; Coropceanu, I.; Kamysbayev, V.; Janke, E. M.; Zajac, M.; Yazdani, N.; Cotts, B. L.; Park, S.; Sood, A.; et al. Nonequilibrium Thermodynamics of Colloidal Gold Nanocrystals Monitored by Ultrafast Electron Diffraction and Optical Scattering Microscopy. *Acs Nano* **2020**, *14* (4), 4792-4804.
